# Incidence and prevalence of neuroendocrine tumors of the lung: analysis of a US commercial insurance claims database

**DOI:** 10.1186/s12890-018-0678-5

**Published:** 2018-08-13

**Authors:** Michael S. Broder, Beilei Cai, Eunice Chang, Maureen P. Neary

**Affiliations:** 1 Partnership for Health Analytic Research, LLC, 280 S. Beverly Dr. Ste. 404, Beverly Hills, CA USA; 2 Novartis Pharmaceuticals Corporation, One Health Plaza, East Hanover, New Jersey USA

**Keywords:** Lung neuroendocrine tumors, Epidemiology, Prevalence, Incidence, Insurance claims

## Abstract

**Background:**

As reported in Surveillance, Epidemiology, and End Results (SEER) data, US incidence and prevalence of neuroendocrine tumors (NET) has increased over recent years. The study objective was to update incidence and prevalence information for lung NET using administrative claims.

**Methods:**

This descriptive epidemiological study used 2009–2014 data from 2 US claims databases: MarketScan and PharMetrics. Patients (18–64 years old) had ≥1 inpatient or ≥ 2 outpatient claims with NET of bronchus or lung, identified by International Classification of Diseases, 9th Revision, Clinical Modification diagnosis codes. Prevalence was number of lung NET patients divided by number of enrollees/year. Incidence was number of patients with a first observed NET diagnosis who were disease-free for 2 years prior, divided by number of enrollees. Age and gender adjustments performed.

**Results:**

The annual number of patients with lung NET identified from 2009 to 2014 ranged from 435 to 796 (MarketScan) and 419–648 (PharMetrics). In MarketScan, there was a 7.4% (95%CI 2.1–13.0; *p* = 0.027) annual percent change (APC) in the age-adjusted incidence for males and 6.8% (− 0.2–14.3; 0.052) for females. In PharMetrics, APC was − 2.9% (− 13.8–9.4; 0.395) for males; 14.7% (− 12.9–51.2; 0.165) for females. In MarketScan, APC in age-adjusted prevalence for males was 9.9% (4.7–15.3; 0.006); 16.2% (11.4-21.1; <.001) for females. For PharMetrics, APCs were 9.5% (2.3–17.2; 0.021) for males; 16.3% (9.6–23.5; 0.002) for females.

**Conclusions:**

From 2009 to 2014 there was a statistically significant increase in age-adjusted lung NET incidence for males in MarketScan, and a statistically significant increase in age-adjusted prevalence for both genders in PharMetrics. Incidence and prevalence changes, to the extent they exist, may be due to better diagnostic methods, increased awareness of NET among clinicians and pathologists, and/or an actual increase in US disease occurrence. Differences in rates across databases are difficult to explain. These results suggest the need for awareness of the clinically effective and safe treatment options available for lung NET patients among healthcare providers.

## Background

Neuroendocrine tumors (NET) comprise a broad family of rare and often slow growing malignancies. NET can develop anywhere in the body and arise from neuroendocrine cells throughout the endocrine system [1, 2]. Approximately one-quarter to one-third of NET occur in the lung [3, 4]. NET secrete peptides and neuroamines that may cause distinct syndromes (e.g., carcinoid syndrome, glucagonoma), in which case they are referred to as “functional” tumors. Clinical presentation depends on the site of the primary tumor and whether or not they are functional. Research on risk factors for lung NET is limited, although the authors of a recent meta-analysis concluded, “family history of cancer is the most relevant risk factor for [NET] development at all investigated sites, followed by BMI and diabetes. Cigarette smoking and alcohol consumption are potential risk factors for selected anatomical sites” [5]. Surgery may be curative in the early stages, but delayed diagnosis is typical.

While rare, the incidence and prevalence of NET appear to be increasing worldwide [4–10]. In a 2008 study using the US Surveillance, Epidemiology, and End Results (SEER) database, the incidence of NET in the US increased from 10.9 cases per million person-years (PMPY) in 1973 to 52.5 PMPY in 2004 [4] and in a 2017 study to 69.8 PMPY in 2012 [10]. Overall NET prevalence was 350 per 1 million in 2004 [4] and 480 per 1 million in 2012 [10]. Only patients with malignant cancers are included in the SEER registries, and the separation of NET into clear-cut benign and malignant categories is not as straightforward as it is for most epithelial malignancies [11]. NET that have not invaded adjacent organs or metastasized may not be immediately labeled as malignant. Thus, many small, benign-appearing tumors may not get included in SEER [4].

The objective of this study was to update incidence and prevalence information for lung NET with non-registry-based data, specifically insurance claims, using additional data beyond what had previously been reported.

## Methods

### Design and data source

This was a descriptive epidemiological study using insurance claims data from January 1, 2009 to December 31, 2014. The data were from two large US commercial claims databases: Truven Health MarketScan Commercial Claims and Encounters Database, and IMS Health PharMetrics. The MarketScan database has information from more than 100 payers of private health insurance for employees and their dependents, covering more than 25 million lives annually. The PharMetrics database is a nonpayer owned integrated claims database of commercial insurers that includes medical and pharmacy claims for more than 70 million unique individuals across the US. Both databases contain de-identified adjudicated medical claims (e.g., inpatient and outpatient services) and pharmacy claims (e.g., outpatient prescriptions).

Payments to providers, healthcare facilities, and pharmacies for the 66% of the US population with commercial insurance are contingent on submission of claims for services [12]. These insurance claims contain information about diagnoses (International Classification of Diseases, 9th Revision, Clinical Modification [ICD-9-CM] diagnosis codes) and procedures (Current Procedural Terminology 4 [CPT-4] and ICD-9-CM procedure codes). Information on each physician visit, medical procedure, hospitalization, drug dispensed, date of service, number of days of medication supplied, test performed, as well as complete payment information, is available for covered individuals from their insurance claims. Available patient demographic information is limited to age, gender, and broad geographic region. An “enrollment” file provides information on each individuals’ dates of coverage—the dates for which we can find their insurance claims. No information is available about individuals for dates outside their dates of enrollment. Information about death, including date or cause, is not available. Privacy restrictions make it impossible to contact patients or review their detailed medical records to obtain additional clinical or demographic information such as health behaviors, tumor size/stage, or race/ethnicity. In the US, individuals may change their insurance coverage over time, and thus the number of individuals enrolled in a given plan changes from year to year. For this study, we were provided the total number of individuals in each database each year, broken down by age and gender. Both databases contain a limited number of individuals ≥65 years old. US individuals ≥65 who have commercial insurance are not representative of the broader age group, a large majority of whom are insured through the Federal Medicare program. Therefore, the analysis was restricted to individuals < 65. Analyses were performed separately using each database and results were compared to check consistency. As the data were de-identified, this study was considered exempt from approval by the Institutional Review Board.

### Cohort selection

Individuals at least 18 years of age were identified as having lung NET if, during a single calendar year, they had at least 1 claim from a hospital setting, or at least 2 claims from the outpatient setting, that included an ICD-9-CM diagnosis code for lung NET (that is, either 209.21 malignant carcinoid tumor of bronchus and lung, or 209.61 benign carcinoid tumor of bronchus and lung). Coding of inpatient claims in the US is usually performed by professional coders and is thus more reliable than claims from the outpatient setting, which may be recorded by a variety of staff with limited clinical training. A limitation of using claims data to estimate disease incidence is the inability to know with certainty that the first diagnosis seen in the data represents the first clinical diagnosis of the condition. Therefore, for incident cases, we required individuals to have been continuously enrolled for 3 years: the specific calendar year of diagnosis and 2 years prior, with no evidence of disease in the prior 2 years. For example, a cohort of individuals identified with lung NET in 2011 must have been enrolled during the entire 2009 to 2011 period, with their lung NET diagnosis in 2011.

### Statistical analysis

For each calendar year, we reported the distribution of patient demographics, summarizing continuous variables with means, and categorical variables with patient counts and percentages. Incidence rate was calculated as the number of individuals with lung NET in a particular year divided by the number of all individuals who were continuously enrolled (that is, for whom we had data for the entire year) across the three-year period (year of diagnosis and 2 prior disease-free years) and reported as per million person-years (PMPY). Prevalence was calculated as the number of lung NET patients in a particular year divided by the total number of individuals continuously enrolled for that calendar year and reported as patients per million. For both incidence and prevalence, rates were reported overall and by sex and age categories (18–24, 25–34, 35–44, 45–54, and 55–64 years). To allow comparisons within genders and between databases over time, we calculated adjusted (gender-specific) rates by standardizing the age distribution for each gender based on the distribution of ages (in those same 4 age categories) from both databases in 2014. Similarly, we calculated overall adjusted rates by standardizing to the age and gender distribution from both databases in 2014. The enrollment requirements for inclusion in the incidence and prevalence denominators differed (3 years of enrollment for incidence and a single calendar year for prevalence), and the denominator drops substantially when the continuous enrollment criteria is added. We believe the underlying US commercially insured population is more similar to the one used for calculating prevalence; therefore, to calculate standardized rates for both incidence and prevalence, we used the age and gender distribution from the prevalent population in 2014.

We used annual percent change (APC) to study trends over time [13, 14]. APC was calculated by least-squares linear regression on a log-linear model to characterize trends in rates over calendar year. With this approach, each rate is assumed to change at a constant percentage of the previous year’s rate. Because each database had a different denominator, results are reported separately by database. All data transformations and statistical analysis were performed using SAS® version 9.4 (SAS Institute, Cary, NC).

## Results

On average, in each year from 2009 to 2014, 631 patients were identified as having lung NET in the MarketScan database. The annual number ranged from 435 in 2009 to low of 435 in 2009 to a high of 796 in 2012. In the PharMetrics database, the range was 419 in 2009 to 648 in 2014, with a mean of 559. In the MarketScan and PharMetrics databases, 65.2 and 64.0% of cases were female, respectively (ranging from 59.8 to 69.4%). More than half of the cases (53.0 to 61.7%) were patients between 55 and 64 years old (Table 1).

**Table 1:** Patients with lung NET, N^a^

	MarketScan						PharMetrics					
	2009	2010	2011	2012	2013	2014	2009	2010	2011	2012	2013	2014
N	435	521	681	796	667	687	419	510	563	598	616	648
Age, year, no. (%)												
18–24	9 (2.1)	6 (1.2)	9 (1.3)	9 (1.1)	7 (1.0)	2 (0.3)	7 (1.7)	5 (1.0)	10 (1.8)	10 (1.7)	7 (1.1)	9 (1.4)
25–34	16 (3.7)	27 (5.2)	26 (3.8)	37 (4.6)	26 (3.9)	30 (4.4)	29 (6.9)	18 (3.5)	23 (4.1)	19 (3.2)	27 (4.4)	17 (2.6)
35–44	44 (10.1)	57 (10.9)	67 (9.8)	75 (9.4)	68 (10.2)	79 (11.5)	38 (9.1)	59 (11.6)	46 (8.2)	61 (10.2)	65 (10.6)	60 (9.3)
45–54	117 (26.9)	152 (29.2)	205 (30.1)	217 (27.3)	182 (27.3)	198 (28.8)	123 (29.4)	136 (26.7)	156 (27.7)	166 (27.8)	165 (26.8)	162 (25.0)
55–64	249 (57.2)	279 (53.6)	374 (54.9)	458 (57.5)	384 (57.6)	378 (55.0)	222 (53.0)	292 (57.3)	328 (58.3)	342 (57.2)	352 (57.1)	400 (61.7)
Female	260 (59.8)	340 (65.3)	445 (65.3)	515 (64.7)	463 (69.4)	459 (66.8)	258 (61.6)	315 (61.8)	352 (62.5)	392 (65.6)	403 (65.4)	435 (67.1)

^a^ Adult patients (age 18 years or older) with ≥1 inpatient or ≥ 2 outpatient claims for lung NET in a calendar year. Patients may have been identified in multiple calendar years. Continuous enrollment not required

Generally, in every year and for both databases, unadjusted incidence was higher for each successive age group. Incidence was highest in the two oldest groups: 12.2–27.8 PMPY (depending on year and gender) in individuals aged 45–54, compared to 25.7–53.6 PMPY in individuals aged 55–64 in MarketScan; and 8.2–19.5 PMPY in individuals 45–54 compared to 20.6–55.4 in 55–64 year olds in PharMetrics (Tables 2 and 3).

**Table 2:** MarketScan Database: Lung NET Incidence Rate, Cases per Million Person-Years^a^

		No. Of Cases Per Million Person-Years (Numerator/Denominator)											
		2011			2012			2013			2014		
Gender	Age												
Female	18–24	0.0	(0	/629,902)	2.6	(2	/761,959)	2.5	(2	/806,972)	0.0	(0	/791,556)
	25–34	5.8	(5	/857,782)	4.4	(4	/905,705)	2.7	(2	/751,148)	1.4	(1	/711,916)
	35–44	10.3	(14	/1,361,165)	7.5	(11	/1,460,802)	12.7	(16	/1,264,584)	11.6	(14	/1,203,355)
	45–54	15.8	(28	/1,767,104)	23.3	(44	/1,889,625)	26.7	(44	/1,645,900)	27.8	(43	/1,547,477)
	55–64	49.2	(76	/1,545,517)	44.5	(75	/1,687,254)	49.0	(75	/1,531,896)	53.6	(78	/1,454,099)
	All Female	20.0	(123	/6,161,470)	20.3	(136	/6,705,345)	23.2	(139	/6,000,500)	23.8	(136	/5,708,403)
Male	18–24	1.6	(1	/632,342)	0.0	(0	/768,240)	0.0	(0	/829,650)	0.0	(0	/813,048)
	25–34	1.3	(1	/741,337)	2.6	(2	/775,911)	6.2	(4	/648,646)	1.6	(1	/626,307)
	35–44	5.7	(7	/1,221,845)	6.2	(8	/1,294,055)	5.4	(6	/1,120,973)	7.5	(8	/1,066,055)
	45–54	12.2	(19	/1,562,471)	13.9	(23	/1,656,616)	14.5	(21	/1,446,886)	16.8	(23	/1,365,158)
	55–64	25.7	(35	/1,363,927)	28.8	(42	/1,458,077)	27.2	(36	/1,323,391)	31.8	(40	/1,256,884)
	All Male	11.4	(63	/5,521,922)	12.6	(75	/5,952,899)	12.5	(67	/5,369,546)	14.0	(72	/5,127,452)
All Gender	18–24	0.8	(1	/1,262,244)	1.3	(2	/1,530,199)	1.2	(2	/1,636,622)	0.0	(0	/1,604,604)
	25–34	3.8	(6	/1,599,119)	3.6	(6	/1,681,616)	4.3	(6	/1,399,794)	1.5	(2	/1,338,223)
	35–44	8.1	(21	/2,583,010)	6.9	(19	/2,754,857)	9.2	(22	/2,385,557)	9.7	(22	/2,269,410)
	45–54	14.1	(47	/3,329,575)	18.9	(67	/3,546,241)	21.0	(65	/3,092,786)	22.7	(66	/2,912,635)
	55–64	38.2	(111	/2,909,444)	37.2	(117	/3,145,331)	38.9	(111	/2,855,287)	43.5	(118	/2,710,983)
	All Patients	15.9	(186	/11,683,392)	16.7	(211	/12,658,244)	18.1	(206	/11,370,046)	19.2	(208	/10,835,855)
		Adjusted Rate (No. Of Cases Per Million Person-Years)^b^											
All Female^c^		18.3			18.6			21.3			21.8		
All Male^d^		10.3			11.4			11.7			12.9		
All Patients^e^		14.4			15.2			16.6			17.5		

^a^ Cases (adults with ≥1 inpatient or ≥ 2 outpatient claims for lung NET in listed year and continuous enrollment in year listed and two years prior) ÷ number of members with continuous enrollment in same period

^b^APC (95% CI; P value): female 6.8% (−0.2–14.3; 0.052); male 7.4% (2.1–13.0; 0.027); all patients 7.0% (4.3–9.8; 0.008)

^c^Adjusted based on distribution of age among male from both databases in 2014

^d^Adjusted based on distribution of age among female from both databases in 2014

^e^Adjusted based on combined distribution of age among male from both databases in 2014

**Table 3:** PharMetrics Database: Lung NET Incidence Rate, Cases per Million Person-Years^a^

		No. Of Cases Per Million Person-Years (Numerator/Denominator)											
		2011			2012			2013			2014		
Gender	Age												
Female	18–24	2.5	(2	/809,900)	1.2	(1	/803,203)	0.0	(0	/844,220)	1.5	(1	/670,429)
	25–34	5.5	(5	/915,829)	4.7	(4	/846,136)	3.5	(3	/851,694)	1.5	(1	/659,964)
	35–44	2.2	(3	/1,387,525)	7.0	(9	/1,280,702)	12.8	(16	/1,249,676)	10.3	(10	/972,515)
	45–54	10.0	(19	/1,902,036)	18.9	(33	/1,747,518)	18.9	(32	/1,688,747)	19.5	(25	/1,282,436)
	55–64	37.6	(68	/1,809,406)	47.4	(81	/1,708,362)	55.4	(94	/1,697,399)	51.9	(67	/1,290,342)
	All Female	14.2	(97	/6,824,696)	20.0	(128	/6,385,921)	22.9	(145	/6,331,736)	21.3	(104	/4,875,686)
Male	18–24	1.2	(1	/828,261)	3.6	(3	/823,432)	2.3	(2	/875,326)	1.4	(1	/698,342)
	25–34	2.4	(2	/839,247)	1.2	(1	/800,680)	0.0	(0	/818,984)	1.5	(1	/662,957)
	35–44	5.5	(7	/1,266,850)	5.1	(6	/1,185,774)	3.4	(4	/1,164,816)	6.4	(6	/935,200)
	45–54	10.5	(18	/1,713,588)	11.9	(19	/1,599,135)	12.8	(20	/1,556,840)	8.2	(10	/1,218,043)
	55–64	28.7	(47	/1,637,294)	20.6	(32	/1,552,538)	23.2	(36	/1,551,338)	26.3	(32	/1,216,691)
	All Male	11.9	(75	/6,285,240)	10.2	(61	/5,961,559)	10.4	(62	/5,967,304)	10.6	(50	/4,731,233)
All Gender	18–24	1.8	(3	/1,638,161)	2.5	(4	/1,626,635)	1.2	(2	/1,719,546)	1.5	(2	/1,368,771)
	25–34	4.0	(7	/1,755,076)	3.0	(5	/1,646,816)	1.8	(3	/1,670,678)	1.5	(2	/1,322,921)
	35–44	3.8	(10	/2,654,375)	6.1	(15	/2,466,476)	8.3	(20	/2,414,492)	8.4	(16	/1,907,715)
	45–54	10.2	(37	/3,615,624)	15.5	(52	/3,346,653)	16.0	(52	/3,245,587)	14.0	(35	/2,500,479)
	55–64	33.4	(115	/3,446,700)	34.7	(113	/3,260,900)	40.0	(130	/3,248,737)	39.5	(99	/2,507,033)
	All Patients	13.1	(172	/13,109,936)	15.3	(189	/12,347,480)	16.8	(207	/12,299,040)	16.0	(154	/9,606,919)
		Adjusted Rate (No. Of Cases Per Million Person-Years)^b^											
All Female^c^		12.8			18.0			20.6			19.3		
All Male^d^		10.6			9.3			9.3			9.6		
All Patients^e^		11.7			13.8			15.2			14.6		

^a^ Cases (adults with ≥1 inpatient or ≥ 2 outpatient claims for lung NET in listed year and continuous enrollment in year listed and two years prior) ÷ number of members with continuous enrollment in same period

^b^APC (95% CI; P value): female 14.7% (−12.9–51.2; 0.165); male −2.9% (−13.8–9.4; 0.395); all patients 7.8% (−5.7–23.4; 0.137)

^c^Adjusted based on distribution of age among male from both databases in 2014

^d^Adjusted based on distribution of age among female from both databases in 2014

^e^Adjusted based on combined distribution of age among male from both databases in 2014

**Fig. 1:**
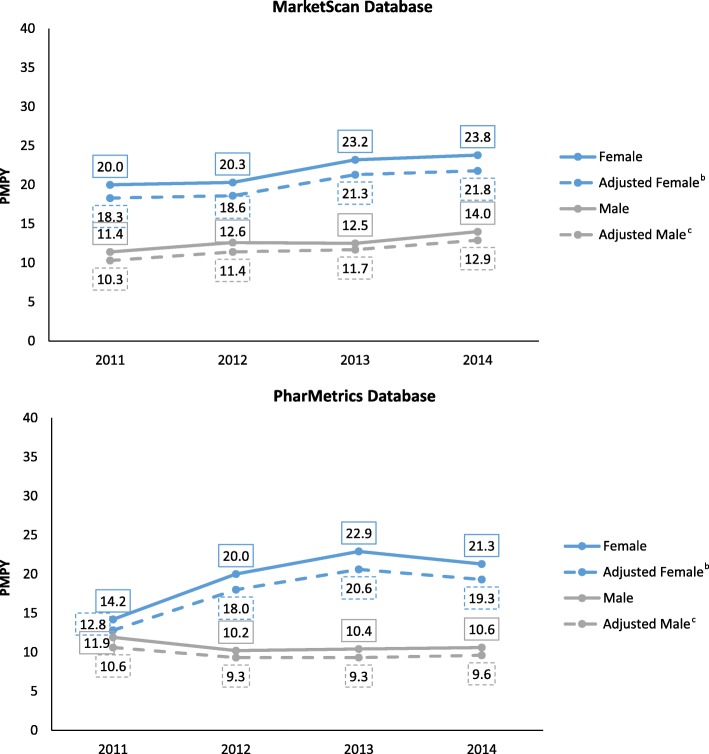
Lung NET Incidence Rate, Cases per Million Person-Years^a^. ^a^ In the combined database (adjusted for age and gender) the no. of cases per million person-years was 13.0 (2011), 14.5 (2012), 15.9 (2013), and 16.2 (2014) for all patients. ^b^ Adjusted based on distribution of age among males from both databases in 2014. ^c^ Adjusted based on distribution of age among females from both databases in 2014

After adjustment for age and gender, in the MarketScan database combined (males and females) incidence increased from 14.4 PMPY in 2011 and to 17.5 in 2014, an annual percent change (APC) (95% CI; *P* value) of 7.0% (4.3–9.8; 0.008). The gender-specific incidence (adjusted for age) showed a statistically significant change for males: 7.4% (2.1–13.0; 0.027); and a similar (but not statistically significant) change for females: 6.8% (− 0.2–14.3; 0.052). In the PharMetrics database, the overall age and gender-adjusted incidence was 11.7 PMPY in 2011, 13.8 in 2012, 15.2 in 2013 and 14.6 in 2014, an APC of 7.8% (− 5.7–23.4; 0.137) (Fig. 1, Tables 2 and 3). The APC was not statistically significant for males (− 2.9% [− 13.8–9.4; 0.395]) or females (14.7% [− 12.9–51.2; 0.165]) individually. When data from MarketScan and PharMetrics were combined and adjusted to the age-gender distribution for 2014, incidence rose from 13.0 PMPY in 2011 to 16.2 PMPY in 2014, an overall APC of 7.7% (1.3–14.4; 0.035). However, the combined result masks differences in results across the 2 databases, as described above.

**Fig. 2:**
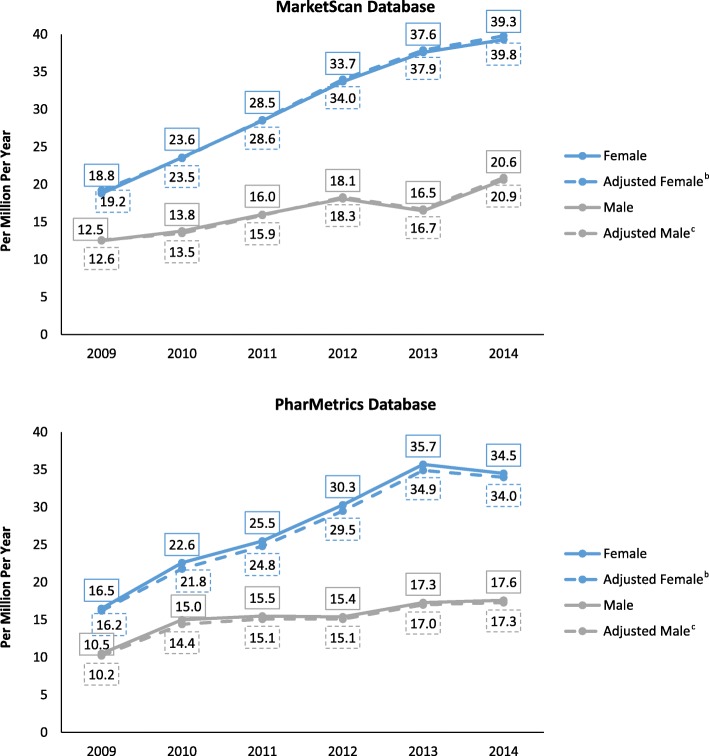
Lung NET Prevalence, Cases per Million/Year^a^. ^a^ In the combined database (adjusted for age and gender) the no. of cases per million per year was 14.6 (2009), 18.4 (2010), 21.3 (2011), 24.6 (2012), 26.9 (2013), and 28.5 (2014) for all patients. ^b^ Adjusted based on distribution of age among males from both databases in 2014. ^c^ Adjusted based on distribution of age among females from both databases in 2014

With few exceptions, in each year and for both databases, unadjusted prevalence was higher for each successive age group. Prevalence was highest in 55–64 year olds (between 25.1 and 102.3 per million, depending on year, gender, and database). With few exceptions, unadjusted prevalence was higher in females than in males in every age category (Tables 4 and 5). After age and gender adjustment, prevalence for males and females combined rose from 16.0 per million in 2009 to 30.7 in 2014 in the MarketScan database, an APC of 14.0% (10.2–17.9; <.001). The age-adjusted APC in prevalence for females was 16.2% (11.4–21.1; <.001) and for males was 9.9% (4.7–15.3; 0.006). The age- and gender-adjusted APC in the PharMetrics database was 13.9% (7.4–20.9; 0.004); 16.3% (9.6–23.5; 0.002) for females and 9.5% (2.3–17.2; 0.021) for males. (Fig. 2, Tables 4 and 5). When both datasets were combined and adjusted for age and gender, prevalence rose from 14.6 cases per million in 2009 to 28.5 per million in 2014, an APC of 14.2% (9.5–19.0; <.001) overall (16.5% [10.9–22.3; 0.001] for females and 9.9% (5.6–14.3; 0.003) for males.

**Table 4:** MarketScan Database: Lung NET Prevalence, Cases per Million per Year^a^

		No. Of Cases Per Million Per Year (Numerator/Denominator)																	
		2009			2010			2011			2012			2013			2014		
Gender	Age																		
Female	18–24	1.7	(2	/1,200,214)	1.7	(2	/1,179,801)	0.7	(1	/1,535,683)	3.0	(5	/1,668,409)	3.6	(5	/1,404,034)	0.7	(1	/1,342,845)
	25–34	4.9	(10	/2,032,535)	8.2	(16	/1,962,108)	5.8	(12	/2,052,889)	10.5	(22	/2,089,096)	9.8	(16	/1,640,542)	8.3	(13	/1,562,298)
	35–44	7.6	(20	/2,616,976)	9.9	(25	/2,533,283)	12.9	(34	/2,643,239)	14.7	(39	/2,660,474)	18.8	(40	/2,123,456)	21.0	(42	/1,996,743)
	45–54	18.1	(56	/3,090,478)	24.7	(75	/3,031,223)	31.0	(99	/3,190,617)	36.8	(118	/3,204,967)	40.3	(102	/2,529,219)	42.4	(99	/2,336,993)
	55–64	52.8	(124	/2,349,482)	59.7	(144	/2,411,232)	75.1	(197	/2,624,303)	85.9	(231	/2,688,474)	95.2	(209	/2,194,982)	102.3	(210	/2,053,379)
	All Female	18.8	(212	/11,289,685)	23.6	(262	/11,117,647)	28.5	(343	/12,046,731)	33.7	(415	/12,311,420)	37.6	(372	/9,892,233)	39.3	(365	/9,292,258)
Male	18–24	5.2	(6	/1,147,214)	3.5	(4	/1,129,804)	2.6	(4	/1,549,206)	2.3	(4	/1,705,487)	1.4	(2	/1,431,673)	0.0	(0	/1,365,613)
	25–34	2.3	(4	/1,760,823)	4.8	(8	/1,669,422)	3.9	(7	/1,788,710)	4.9	(9	/1,850,139)	4.2	(6	/1,436,317)	5.8	(8	/1,388,742)
	35–44	7.2	(17	/2,359,613)	7.1	(16	/2,250,466)	11.3	(27	/2,381,647)	9.2	(22	/2,403,534)	8.9	(17	/1,900,182)	12.3	(22	/1,791,028)
	45–54	10.3	(28	/2,731,422)	15.9	(42	/2,636,290)	16.7	(47	/2,817,734)	21.1	(60	/2,841,640)	18.4	(41	/2,223,287)	24.2	(50	/2,066,977)
	55–64	33.7	(71	/2,104,573)	30.8	(65	/2,107,551)	38.3	(88	/2,296,348)	45.7	(107	/2,342,101)	42.7	(81	/1,897,684)	52.1	(93	/1,783,325)
	All Male	12.5	(126	/10,103,645)	13.8	(135	/9,793,533)	16.0	(173	/10,833,645)	18.1	(202	/11,142,901)	16.5	(147	/8,889,143)	20.6	(173	/8,395,685)
All Gender	18–24	3.4	(8	/2,347,428)	2.6	(6	/2,309,605)	1.6	(5	/3,084,889)	2.7	(9	/3,373,896)	2.5	(7	/2,835,707)	0.4	(1	/2,708,458)
	25–34	3.7	(14	/3,793,358)	6.6	(24	/3,631,530)	4.9	(19	/3,841,599)	7.9	(31	/3,939,235)	7.2	(22	/3,076,859)	7.1	(21	/2,951,040)
	35–44	7.4	(37	/4,976,589)	8.6	(41	/4,783,749)	12.1	(61	/5,024,886)	12.0	(61	/5,064,008)	14.2	(57	/4,023,638)	16.9	(64	/3,787,771)
	45–54	14.4	(84	/5,821,900)	20.6	(117	/5,667,513)	24.3	(146	/6,008,351)	29.4	(178	/6,046,607)	30.1	(143	/4,752,506)	33.8	(149	/4,403,970)
	55–64	43.8	(195	/4,454,055)	46.3	(209	/4,518,783)	57.9	(285	/4,920,651)	67.2	(338	/5,030,575)	70.9	(290	/4,092,666)	79.0	(303	/3,836,704)
	All Patients	15.8	(338	/21,393,330)	19.0	(397	/20,911,180)	22.6	(516	/22,880,376)	26.3	(617	/23,454,321)	27.6	(519	/18,781,376)	30.4	(538	/17,687,943)
		Adjusted Rate (No. Of Cases Per Million Per Year)^b^																	
All Female^c^		19.2			23.5			28.6			34.0			37.9			39.8		
All Male^d^		12.6			13.5			15.9			18.3			16.7			20.9		
All Patients^e^		16.0			18.7			22.5			26.4			27.6			30.7		

^a^ Cases of adults with ≥1 inpatient or ≥ 2 outpatient claims for Lung NET in listed year and continuous enrollment in year listed ÷ number of members with continuous enrollment in same period

^b^APC (95% CI; P value): female 16.2% (11.4–21.1; <.001); male 9.9% (4.7–15.3; 0.006); all patients: 14.0% (10.2–17.9; <.001)

^**c**^Adjusted based on distribution of age among males from both databases in 2014

^d^Adjusted based on distribution of age among females from both databases in 2014

^e^Adjusted based on combined distribution of age among males from both databases in 2014

**Table 5:** PharMetrics Database: Lung NET Prevalence, Cases per Million per Year^a^

		No. Of Cases Per Million Per Year (Numerator/Denominator)																	
		2009			2010			2011			2012			2013			2014		
Gender	Age																		
Female	18–24	2.9	(4	/1,388,059)	2.3	(3	/1,318,141)	3.5	(5	/1,431,236)	4.4	(6	/1,360,555)	2.2	(3	/1,335,252)	1.9	(2	/1,048,351)
	25–34	6.6	(13	/1,960,213)	3.8	(7	/1,849,997)	8.1	(15	/1,851,913)	4.7	(8	/1,708,390)	7.1	(12	/1,684,490)	3.7	(5	/1,341,604)
	35–44	6.0	(15	/2,508,808)	10.7	(25	/2,335,199)	6.6	(15	/2,269,096)	12.2	(25	/2,055,088)	20.3	(40	/1,969,715)	16.1	(25	/1,553,850)
	45–54	18.7	(59	/3,149,529)	24.0	(71	/2,959,455)	26.5	(76	/2,867,408)	31.9	(82	/2,574,529)	32.8	(80	/2,436,504)	35.2	(66	/1,875,797)
	55–64	38.2	(101	/2,643,692)	55.5	(143	/2,576,843)	65.6	(169	/2,575,456)	77.4	(184	/2,377,377)	92.0	(212	/2,305,347)	91.9	(165	/1,796,085)
	All Female^b^	16.5	(192	/11,650,301)	22.6	(249	/11,039,635)	25.5	(280	/10,995,109)	30.3	(305	/10,075,939)	35.7	(347	/9,731,308)	34.5	(263	/7,615,687)
Male	18–24	1.4	(2	/1,387,054)	1.5	(2	/1,322,440)	1.4	(2	/1,462,310)	2.8	(4	/1,406,293)	2.9	(4	/1,386,541)	3.6	(4	/1,097,662)
	25–34	4.5	(8	/1,759,919)	3.0	(5	/1,671,459)	3.0	(5	/1,687,428)	1.9	(3	/1,609,181)	6.2	(10	/1,604,977)	2.3	(3	/1,323,432)
	35–44	4.3	(10	/2,300,612)	12.6	(27	/2,147,076)	10.5	(22	/2,093,812)	9.3	(18	/1,928,820)	10.2	(19	/1,864,293)	10.6	(16	/1,505,314)
	45–54	11.2	(32	/2,850,321)	14.9	(40	/2,681,967)	16.1	(42	/2,602,786)	20.2	(48	/2,375,529)	19.4	(44	/2,267,225)	17.9	(32	/1,786,707)
	55–64	25.1	(60	/2,392,616)	33.5	(78	/2,331,410)	37.4	(87	/2,325,581)	33.8	(73	/2,158,306)	39.3	(83	/2,110,883)	44.5	(75	/1,684,375)
	All Male^c^	10.5	(112	/10,690,522)	15.0	(152	/10,154,352)	15.5	(158	/10,171,917)	15.4	(146	/9,478,129)	17.3	(160	/9,233,919)	17.6	(130	/7,397,490)
All Gender	18–24	2.2	(6	/2,775,113)	1.9	(5	/2,640,581)	2.4	(7	/2,893,546)	3.6	(10	/2,766,848)	2.6	(7	/2,721,793)	2.8	(6	/2,146,013)
	25–34	5.6	(21	/3,720,132)	3.4	(12	/3,521,456)	5.7	(20	/3,539,341)	3.3	(11	/3,317,571)	6.7	(22	/3,289,467)	3.0	(8	/2,665,036)
	35–44	5.2	(25	/4,809,420)	11.6	(52	/4,482,275)	8.5	(37	/4,362,908)	10.8	(43	/3,983,908)	15.4	(59	/3,834,008)	13.4	(41	/3,059,164)
	45–54	15.2	(91	/5,999,850)	19.7	(111	/5,641,422)	21.6	(118	/5,470,194)	26.3	(130	/4,950,058)	26.4	(124	/4,703,729)	26.8	(98	/3,662,504)
	55–64	32.0	(161	/5,036,308)	45.0	(221	/4,908,253)	52.2	(256	/4,901,037)	56.7	(257	/4,535,683)	66.8	(295	/4,416,230)	69.0	(240	/3,480,460)
	All Patients^d^	13.6	(304	/22,340,823)	18.9	(401	/21,193,987)	20.7	(438	/21,167,026)	23.1	(451	/19,554,068)	26.7	(507	/18,965,227)	26.2	(393	/15,013,177)
		Adjusted Rate (No. Of Cases Per Million Per Year)^b^																	
All Female^c^		16.2			21.8			24.8			29.5			34.9			34.0		
All Male^d^		10.2			14.4			15.1			15.1			17.0			17.3		
All Patients^e^		13.3			18.2			20.1			22.6			26.3			25.9		

^a^ Cases of adults with ≥1 inpatient or ≥ 2 outpatient claims for Lung NET in listed year and continuous enrollment in year listed ÷ number of members with continuous enrollment in same period

^b^APC (95% CI; P value): female 16.3% (9.6–23.5; 0.002); male 9.5% (2.3–17.2; 0.021); all patients: 13.9% (7.4–20.9; 0.004)

^c^Adjusted based on distribution of age among males from both databases in 2014

^d^Adjusted based on distribution of age among females from both databases in 2014

^e^Adjusted based on combined distribution of age among males from both databases in 2014

## Discussion

From 2009 to 2014 there was a statistically significant increase in age-adjusted lung NET incidence for males in the MarketScan database. Incidence increased at an annual age and gender-adjusted rate of 7.0% per year, reaching an overall 17.5 PMPY by 2014. In the same database, prevalence rose at an annual age- and gender-adjusted rate of 14.0% per year, reaching 30.7 cases per million per year. The number of cases identified, incidence, and prevalence were all higher in the MarketScan database than in the PharMetrics database. In the PharMetrics database, incidence increased 7.8% per year, but this change was not statistically significant, while age- and gender-adjusted prevalence increased 13.9% per year (*p* < .001). A study using older data recently reported the incidence of NET in the US increased from 3 cases PMPY in 1973 to 16 PMPY in 2012 [10]. The preponderance of female cases has been observed in prior studies using these and other data sources [4, 9, 15].

There are many possible reasons for the observed changes in incidence and prevalence, although determining which reason or reasons are most important was beyond the scope of this study. First, more tumors may be found incidentally over time. Rates of both CT and MRI use in the general population have been steadily increasing, as has the accuracy of these tests [16]. Some patients may have their tumors detected simply because they have a chest imaging study for another reason. Second, screening for lung cancer appears to be rising [17]. As screening rates increase, more lung NET may be detected. Third, in the last decade, high sensitivity assays for 5-hydroxyindoleacetic acid (5-HIAA) and chromogranin A, both markers for certain NET, have come into more common use. Increased use of these tests may have improved identification of previously undetected tumors [18]. Fourth, pathologists may be improving their ability to identify NET. Finally, the underlying rate of the development of NET may be increasing. The increased prevalence would be expected from the combination of increasing incidence [4, 10] and improved survival [19, 20]. Incidental identification of lung NET might also partially explain the finding that prevalence increased more than incidence during the period studied. If earlier tumors were being identified, survival would appear to increase, which in turn would increase prevalence. We could not test this theory in the current study, as data on disease stage is lacking. A recent analysis of pancreatic NET using the SEER database found increases in both incidence and survival and concluded that stage migration, or an increased detection of localized disease, explained at least part of these observations [21]. In the current study, although the adjusted annual percent change differed between the PharMetrics and MarketScan databases, these between-database differences were not statistically significant (e.g., the 95% CI overlapped) and the estimate from the combined databases was consistent with the individual ones. Both databases are derived from wide geographic regions, encompass diverse practice types, and represent multiple insurance plan types, between-database differences on any of these individual factors may explain why the estimates are numerically different between PharMetrics and MarketScan.

Estimates from the current study are consistent with that reported from SEER for 2012 [10]. The incidence of all NET was reported as 69.8 PMPY for 2012 and lung NET as 16.3, and our combined, adjusted estimate was 16.2 PMPY in 2014 (although this rate from the combined databases should be interpreted with caution since it obscures differences between different sources). Although these numbers are quite similar, comparing our results directly to prior estimates presents several challenges. First, we were able to use data through 2014, 2 years more recent than SEER. Second, SEER, the source of data for the 2008 and 2017 studies, is a coordinated system of population-based cancer registries located across the US. The SEER Program collects cancer incidence and survival data from 18 geographic areas, together representing about 1/4 of the US population [22]. The insurance claims used in the present study, in contrast, are a convenience sample, albeit an extremely large one. Based on information provided by MarketScan and PharMetrics, the combined databases have claims for a geographically dispersed sample representing about 1/3 of the US population. Third, SEER includes patients of all ages; the current study only included individuals 18–64 years of age. About 95% of individuals ≥65 are covered by Medicare [12]. A small number of individuals in this age group would have been available for inclusion in our study (e.g. they had commercial insurance as the primary payer, and therefore, their data were included in our databases), but they do not represent typical Medicare-age individuals, and thus were excluded from analysis. The incidence of NET is twice as high in individuals ≥65 compared to those 50–64 [10]. Our estimates are thus likely to understate the actual incidence and prevalence. Fourth, SEER registrars are trained and provided with software to improve their ability to accurately code reportable cancers. Claims coding is performed by a mix of care providers and professional coders. Fourth, the coding systems differ between SEER and insurance claims. SEER currently uses the International Classification of Diseases for Oncology system (ICD-O-3), whereas claims use ICD-9-CM (since 2015, ICD-10). While the systems can be mapped to each other, the mapping is not one-to-one. NET represent an unusual tumor type for which the traditional labels of benign and malignant are a poor fit. While classification has evolved considerably in the last several decades, NET are now generally described by their anatomic location (e.g., GI or lung), degree of differentiation (either “well” or “poorly”), and proliferative index (mitotic activity). Small, well differentiated NET may thus have been overlooked for inclusion into SEER [4]. Insurance claims, relying as they do on ICD-9-CM codes, cannot be used to identify stage or tumor size. Claims data cannot be used to identify with certainty whether a case is truly incident or represents a patient who simply did not present for care for a prolonged period. We required continuous enrollment for 2 years before the first NET claim to reduce this source of uncertainty. Prevalent patients would have to have had no care for their condition for more than 2 years to have been incorrectly counted as incident cases. Other limitations of insurance claims include the lack of information on race/ethnicity or health behaviors that might explain the observed rise in lung NET. Finally, we had no information on occurrence of, timing, or cause of death, making it impossible to comment on survival.

Despite these differences and data limitations, both the prior SEER study and the current study have identified some, although not entirely consistent, evidence of increasing incidence and prevalence of lung NET. The consistent pattern in 3 databases over more than 4 decades strongly suggests the increase in lung NET cases is not an artifact of the database chosen, the method used, or changes in patient enrollment over time. Recent increases in other cancer types have a variety of explanations. At least some portion of the recent increases in thyroid cancer appears to result from improved screening [23], but there also appears to be an underlying increase in disease incidence as well [24]. In NET, multiple mechanisms may be operating, and studies using other sources of data will be required to determine the extent to which each contributes to the observed rise.

## Conclusions

The incidence and prevalence of lung NET appears to be increasing over time, although in the current study the gender- and database-specific findings are inconsistent. Although lung cancer overall appears to be on the decline in the US [25], an increase in NET in multiple anatomic locations, including lung NET, has been observed [10]. Pulmonologists, gastroenterologists, oncologists, and other physicians may see patients with these tumors with increasing frequency in years to come and may thus need to become more familiar with its presentation and treatment. Health plans will also see an increase in this previously rare disease and should consider ways to effectively manage this population. Finally, because higher incidence brings higher costs, studies assessing the increasing economic burden of this disease are warranted.

## Abbreviations

5-HIAA: 5-hydroxyindoleacetic acid
APC: annual percent change
CPT-4: Current Procedural Terminology 4
ICD-9-CM: International Classification of Diseases, 9th Revision, Clinical Modification
ICD-O-3: International Classification of Diseases for Oncology system
NET: Neuroendocrine tumors
PMPY: Per million person-years
SEER: Surveillance Epidemiology, and End Results
